# Recombinant *Lactococcus Lactis* Expressing M1-HA2 Fusion Protein Provides Protective Mucosal Immunity Against H9N2 Avian Influenza Virus in Chickens

**DOI:** 10.3389/fvets.2020.00153

**Published:** 2020-03-24

**Authors:** Zhou Sha, Hongqi Shang, Yongqiang Miao, Jin Huang, Xiangyun Niu, Ruichang Chen, Liping Hu, He Huang, Kai Wei, Ruiliang Zhu

**Affiliations:** ^1^Shandong Provincial Key Laboratory of Animal Biotechnology and Disease Control and Prevention, Shandong Agricultural University, Tai'an, China; ^2^Shandong Provincial Engineering Technology Research Center of Animal Disease Control and Prevention, Shandong Agricultural University, Tai'an, China; ^3^Animal Disease Prevention and Control Center of Shandong Province, Animal Husbandry and Veterinary Bureau of Shandong Province, Jinan, China; ^4^Shandong New Hope Liuhe Co., Ltd, New Hope Group, Qingdao, China

**Keywords:** H9N2, immune responses, *Lactococcus lactis*, oral vaccine, mucosal immune

## Abstract

H9N2 subtype low pathogenicity avian influenza virus (LPAIV) is distributed worldwide and causes enormous economic losses in the poultry industry. Despite immunization of almost all chickens with inactivated vaccines, the disease still remains widespread. We speculated that improving mucosal or cellular immune responses could contribute to improved control of H9N2 viruses. In this study, we constructed a novel *Lactococcus lactis* (*L. lactis*) strain expressing a recombinant fusion protein consisting of the M1 and HA2 proteins derived from an antigenically conserved endemic H9N2 virus strain. The M1-HA2 fusion protein was cloned downstream of a gene encoding a secretory peptide, and we subsequently confirmed that the fusion protein was secreted from *L. lactis* by Western blotting. We assessed the immunogenicity and protective effects of this recombinant *L. lactis* strain. Eighty 1-day-old chickens were divided into four groups, and the experimental groups were orally vaccinated twice with the recombinant *L. lactis* strain. Fecal and intestinal samples, sera, and bronchoalveolar lavage fluid were collected at 7, 14, and 21 days post-vaccination (dpv). Chickens vaccinated with the recombinant *L. lactis* strain showed significantly increased levels of serum antibodies, T cell-mediated immune responses, and mucosal secretory IgA (SIgA). Following challenge with H9N2 virus at 21 dpv, chickens vaccinated with the recombinant *L. lactis* strain showed decreased weight loss, lower viral titers in the lung, and reduced lung pathological damage. In summary, our results demonstrated that a recombinant *L. lactis* strain expressing an H9N2 M1-HA2 fusion protein could induce protective mucosal and systemic immunity. This oral vaccine is H9N2 virus-specific and represents a significant design improvement compared with previous studies. Our study provides a theoretical basis for improving mucosal immune responses to prevent and control H9N2 virus infection.

## Introduction

Avian influenza virus (AIV) is an important pathogen causing respiratory diseases (1, 2) and severe economic loss to poultry farming (3), and is a serious menace to human health (4). The H9N2 subtype of the low pathogenic avian influenza virus (LPAIV) was first discovered in the United States in 1966. It appeared in China in 1992 and is now widely prevalent worldwide. The H9N2 subtype is still the most important AIV. Infection with this subtype of AIV can result in symptoms like respiratory diseases and decreased egg production in poultry, and may promote a few bacterial infections, leading to high mortality (5). Moreover, H9N2 AIV can directly infect humans and other mammal (6, 7). Studies have reported that the virus could be a gene donor of the other subtypes of AIV, like H5N1 (8). In recent years, the H9N2 AIV has played an increasingly important role in the production of new influenza viruses, such as the H7N9 subtype AIV that has been widely spread in China since 2013, the H10N8 subtype AIV, which infected humans in Jiangxi Province, as well as the H5N2, H5N6, and H5N8 subtypes that are currently prevalent in the poultry industry of China. Additionally, sequence analysis indicates that these internal genes are mainly derived from the H9N2 subtype AIV (9), which highlights the role of H9N2 in the influenza virus ecosystem.

Prevention of H9N2 avian influenza in China is mainly through vaccination with the inactivated vaccine. However, the inactivated vaccines are not able to achieve the desired protective effect; the possible reasons could be low immunization frequency or unreasonable immunization procedure in clinical practice, and the antigenic variation of the epidemic strains, leading to unsatisfactory vaccine protection. Therefore, increasing cellular or mucosal immunity might be crucial to alleviate the limitations of the inactivated vaccine immunization. H9N2 virus is mainly transmitted through the respiratory mucosal pathway, which plays an important role in controlling avian influenza infection; however, conventional vaccines face difficulties in inducing mucosal immunity through the mucosal route. Thus, oral vaccines, which can simultaneously induce mucosal immunity and systemic immunity, might be a viable solution for the prevention and control of the H9N2 virus.

As a food-grade, and thus, safe bacteria, *L. lactis* is widely used as an oral vaccine carrier. As a probiotic, *L. lactis* that are naturally present in the intestines of humans and animals, provide certain nutrients to the body, inhibit the reproduction of harmful bacteria, maintain the balance of the gastrointestinal flora, and thus, improve the function of the gastrointestinal tract (10). Furthermore, *L. lactis* can also stimulate immune cells, enhance the mucosal immune machinery, and naturally activate the immune response (11–13). Many studies have shown that the recombinant expression of viral antigens by *L. lactis* can induce effective antiviral immune responses, such as expression of exogenous severe acute respiratory syndrome (SARS) virus structural proteins, foot-and-mouth disease virus, rotavirus VP7 and VP8 proteins, porcine circovirus type 2 nucleocapsid protein, infectious bursal disease virus VP2 and VP3 proteins, and avian hepatitis E virus (14–17). *In vivo* experiments have shown that the foreign proteins are capable of inducing specific mucosal, humoral, and cellular immune responses and enhancing the ability of an animal to resist pathogen infection. Therefore, it would be advantageous to use *L. lactis* as a carrier to express foreign antigenic proteins.

The viral surface glycoprotein hemagglutinin (HA) is an important immunoprotective antigen of H9N2 virus, which produces specific neutralizing antibodies in different animal models. Hemagglutinin absorbs cellular receptors, like sialic acid, in the host to mediate viral adsorption, transmembrane, and infection. The HA monomer can be cleaved to form HA1 and HA2, wherein the globular structure formed by HA1 is rich in receptor binding sites and has strong variability to recognize most neutralizing antibodies. The stem structure of HA2 is anchored in the viral envelope, and its primary function is to fuse the viral envelope with the cell membrane and release the nucleocapsid of the virion. In addition, the sequence of HA2 is highly conserved, especially in the same subtype; therefore, a vaccine based on the conserved region of HA2 is being considered. The AIV Section 7 RNA encodes matrix protein (M), including M1 and M2 proteins. M1 is responsible for the maintenance of the highly conserved structural protein of AIV that consists of 252 amino acid residues and accounts for 30–40% of the total viral proteins. The M1 protein is involved in many aspects of the viral life cycle and is one of the major target proteins for host T cell recognition during viral infection. Berthoud et al. used the vaccinia vector to express the influenza virus M1 gene, and the clinical trial results showed that the vaccine could induce a strong specific CD8^+^ T lymphocyte reaction in the body (18). Okuda et al. found that the M1 protein-based nucleic acid vaccine not only protected against infection with the homologous influenza virus but also protected against infection with the heterologous influenza virus (19). Therefore, the M1 protein is a potential candidate for the development of cross-protective vaccines.

Controlling the spread of H9N2 subtype avian influenza virus (AIV) may require the development of novel types of vaccines in addition to inactivated vaccines. Oral vaccines are typically able to activate mucosal and systemic immunity, unlike inactivated vaccines. In this study, we identified a clinical isolate of H9N2 subtype AIV with good immunogenicity, and then the M1 and HA2 genes from this isolate as an in-frame fusion was cloned to construct a recombinant *Lactococcus lactis* strain expressing this fusion protein. We evaluated the immunogenicity of this recombinant *L. lactis* strain and its protective effects against H9N2 virus challenge following oral immunization. This study aims to provide a complementary approach for the prevention and control of H9N2 subtype AIV.

## Materials and Methods

### Ethics Statement

The animal experiments were approved by Animal Protection and Utilization Committee of Shandong Agricultural University (Permit number: 20010510) and executed in accordance with Guide to Animal Experiments of Ministry of Science and Technology (Beijing, China). This study did not involve any endangered or protected species.

### Reagents and Strains

The H9N2 virus isolates (A/Chicken/Shandong/01/2018, A/Chicken/Shandong/03/2018, A/Chicken/Shandong/04/2018, A/Chicken/Shandong/06/2018, and A/Chicken/Shandong/11/2017), two commercial H9N2 strains (A/Chicken/Shandong/LG1/2000 and A/Chicken/Shanghai/F/98), and *L. lactis* NZ9000 strain were preserved in our laboratory. HA2 polyclonal antibodies and all positive sera for serum cross-reactivity were prepared by laboratory. *L. lactis* was cultured in GM medium (M17 broth with 0.5% lactose or glucose, optional 1.5% agar). The monoclonal anti-chicken CD4 and CD8 antibodies were purchased from Southern Biotech (San Diego, CA, USA). The H9N2 inactivated vaccine (A/Chicken/Shandong/6/96) was purchased from Biotech Company (Weike Biotechnology, China).

### Construction of Recombinant Expression Vector

Prior to this study, we first assessed serum cross-reactivity against different H9N2 strains isolated in our laboratory. Two commercial strains (LG1/2000 and F/98) served as controls (Table 1). Strain A/Chicken/Shandong/06/2018 was recognized by many sera and was selected as the vaccine candidate strain. The M1 and HA2 genes of this strain were sequenced, and an in-frame fusion gene was synthesized after codon optimization for *L. lactis* (GENEWIZ, China). The fusion protein encoded M1 and HA2 linked by a ten-amino acid linker (Gly_4_Ser-Gly_4_Ser). The gene with Nco I and Xba I restriction sites was cloned into the pNZ8148 vector, which was confirmed by polymerase chain reaction (PCR) and sequencing (TSINGKE, Beijing). The recombinant plasmid pNZ8148-M1-HA2 was transformed into *E. coli* MC1061 strain to expand the yield. The primers used for PCR are listed in Table 2.

**Table 1:** Results of serum cross-reactivity.

**Strains**	HA titer^*^	HI titer^*^						
		**06/2018**	**04/2018**	**03/2018**	**01/2018**	**11/2017**	**LG1**	**F**
A/Chicken/Shandong/06/2018	7	–	6	6	6	5	7	7
A/Chicken/Shandong/04/2018	8	7	–	7	5	6	8	7
A/Chicken/Shandong/03/2018	6	7	6	–	5	5	7	7
A/Chicken/Shandong/01/2018	7	6	5	6	–	6	7	8
A/Chicken/Shandong/11/2017	5	8	5	5	6	–	8	8

* *The results of HA and HI titers are expressed on a log_2_ scale*.

**Table 2:** The primers used in this study.

**Primer name**	Sequence (5^**′**^-3^**′**^)^*^
M1-HA2-F	CCCATGGGTAAAAAAAAGATTAT
M1-HA2-R	CTCTCTAGATTAATGGTGATGATG
8148-F	ACGCGAGCATAATAAACGG
8148-R	CGAAAGCGAAATCAAACGA
IL-2-F	CTCGGAGCTCTGCAGCGTGT
IL-2-R	TCCACCACAGTTGCTGGCTCATC
IL-4-F	CCACGGAGAACGAGCTCATC
IL-4-R	GAGAACCCCAGACTTGTTCTTCA
IFN-γ-F	ACAACCCACAGATCCAGC
IFN-γ-R	TCAGCACCGACTCCTTTT

* *The italic bases encode Nco I (CCATGG) and Xba I (TCTAGA) restriction sites*.

### Expression, Purification, and Characterization of Recombinant M1-HA2 Fusion Protein

Competent *L. lactis* NZ9000 strain was transformed with the recombinant plasmid, yielding the M1-HA2 *L. lactis* strain. The NZ9000 strain was transformed with empty pNZ8148 plasmid yielding the pNZ8148 *L. lactis* strain. Protein expression was induced using nisin. *L. lactis* cells were harvested by centrifugation 12 h post-nisin induction. The culture medium was concentrated using a centrifugal filter device (100 kDa) to collect secreted proteins. Recombinant proteins were identified by Western blotting as described previously (20). All media were prepared according to the Nisin-Controlled Expression System manual.

### Effect of Recombinant Plasmid on Growth Curve of *L. lactis in vitro*

M1-HA2-*L. lactis* strain, pNZ8148-*L. lactis* strain, and the primitive *L. lactis* strain were cultured in the GM medium and allowed to stand for 48 h. The culture medium was taken every 4 h, and the number of viable cells in the medium was determined by using the plate counting method.

### Vaccination and Challenge of Chickens

**Figure 1:**
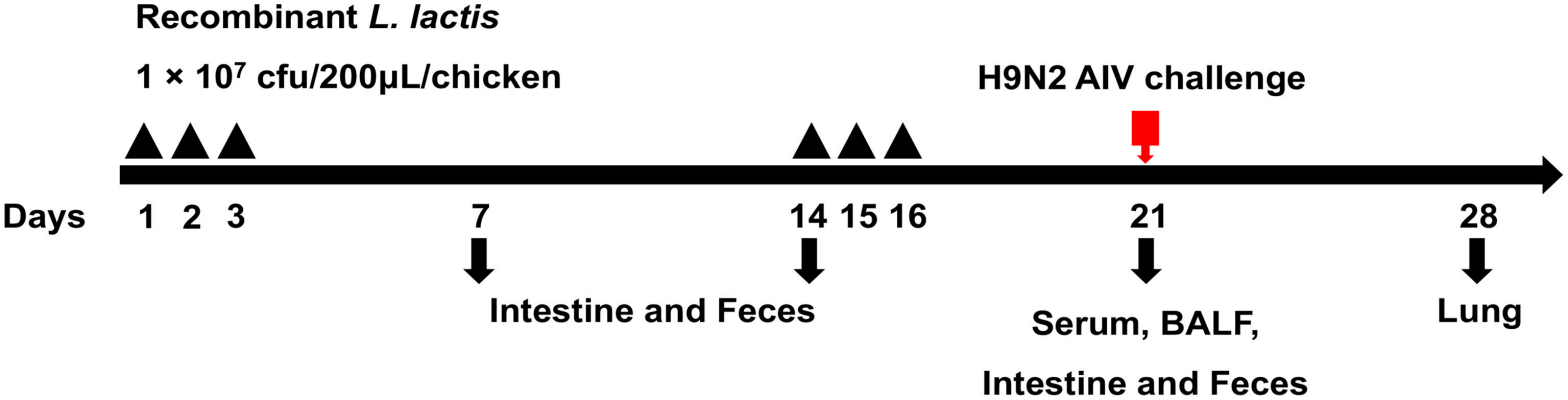
Strategy for the animal experiment. Chickens were grouped as described in the section“Materials and Methods” and orally vaccinated with a priming immunization on days 1, 2, and 3 and with a booster immunization on days 14, 15, and 16. Samples were collected at 7, 14, and 21 dpv for SIgA, IgG, cytokine, and HI titers detection. Seven days after the challenge, samples from the lungs were obtained to evaluate the pathology and virus titer.

Eighty 1-day-old specific-pathogen-free (SPF) chickens were randomly divided into four groups (20 chickens per group). Chickens in the first and second groups were orally vaccinated with 10^7^ cfu/mL of the M1-HA2 and pNZ8148 *L. lactis* strains, respectively. Chickens in the third group were orally vaccinated with phosphate-buffered saline (PBS; 0.2 mL/chicken), and chickens in the fourth group received intramuscular injections of H9N2 inactivated vaccine (0.2 mL/chicken). All chickens received a boosting immunization 1 week later, and then all groups were intranasally challenged with 10^6.0^ EID_50_ of AIV (A/Chicken/Shandong/06/2018). The study procedure is shown in Figure 1.

### Hemagglutination Inhibition (HI) Assay

Sera were collected at 21 dpv. The HI antibody titers of serum antibodies were detected following the previous study (21).

### Mucosal SIgA and Serum IgG Antibodies Detection

The SIgA detection was performed using the Chicken SIgA ELISA Kit (Lengton Bioscience Co., LTD, Shanghai, China), according to the manufacturer's protocol for intestinal tissues. Serum-specific IgG antibody levels were measured by the indirect enzyme-linked immunosorbent assay (ELISA) method, according to a previously reported method (22).

### Real-Time PCR Assays for Detection of Intestinal Mucosal Cytokines

Intestinal mucosal cytokines IL-2, IL-4, and IFN- γ were detected by real-time PCR. Real-time PCR was performed according to the method described by Wang et al. (23). The experimental primers are listed in Table 2. The 2^−ΔΔCT^ method was used to normalize the data (24).

### Measurement of CD4^+^ and CD8^+^ T Lymphocyte Counts in Peripheral Blood

The percentage of CD4^+^ and CD8^+^ T lymphocytes in peripheral blood was measured by flow cytometry, following the method described in a previously published literature from our laboratory (20).

### Analysis of Protective Immune Responses

Seven days following challenge with A/Chicken/Shandong/06/2018, five randomly-selected chickens from each group were sacrificed. Lung tissues were collected, and the viral titers in the lungs were quantitated. Pathological alveolitis and peribronchiolar inflammation in the lungs was visualized by hematoxylin and eosin staining. Changes in body weights of each chicken following challenge were recorded.

### Statistical Analysis

Results were expressed as means ± standard deviation (SD) and analyzed using SPSS 23.0 software (SPSS Incorporated, Chicago, IL, USA). Group differences were compared with Student's *t*-test. *P*-value of 0.05 (*P* ≤ 0.05) was considered statistically significant, and those of 0.01 and below were considered highly statistically significant.

## Results

### Construction, Expression, and Characterization of Recombinant Proteins

**Figure 2:**
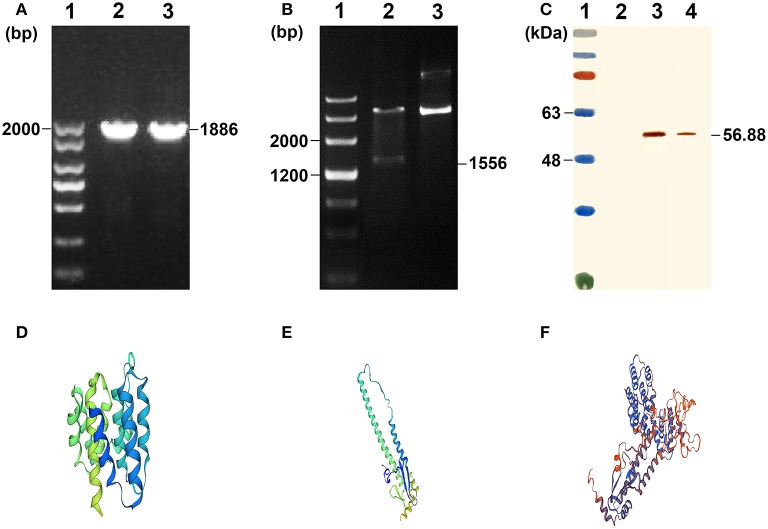
Characterization of the recombinant *L. lactis*. **(A)** Agarose gel results of M1-HA2 gene PCR. Lane 1 is the DNA marker of 2,000 bp; Lane 2-3 are the results for M1-HA2 gene PCR. **(B)** Double digests result of M1-HA2 gene. Lane 1 is the DNA marker of 4,500 bp; Lane 2 is the result for M1-HA2 double digests; Lane 3 is the electrophoresis result of plasmid pNZ8148-M1-HA2. **(C)** Western blotting identification of the recombinant proteins with the rabbit anti–chicken HA2 antibody. Lane 1 is protein molecular size page ruler; Lane 2 is the strain of pNZ8148-*L. lactis*; Lane 3 is the strain of M1-HA2-*L. lactis* at 12 h post induction; Lane 4 is culture supernatant of M1-HA2-*L. lactis* at 12 h post induction. **(D)** 3D structure of the M1 protein; **(E)** 3D structure of the HA2 protein; **(F)** 3D structure of the fusion protein.

The M1-HA2 gene was amplified by PCR, and the product was cloned into the expression vector pNZ8148, which was verified by pNZ8148 universal primers (Figure 2A) and double digestion (Figure 2B). Subsequently, the recombinant pNZ8148-M1-HA2 and blank pNZ8148 plasmids were separately transformed into competent *L. lactis* NZ9000 cells. Upon induction with nisin for 12 h, the novel expressed protein bands corresponding to 56.88 kDa in the concentrated culture supernatants and recombinant pNZ8148-M1-HA2 transformants were visualized through Western blotting analysis (Figure 2C). The result indicated the expression of the recombinant M1-HA2 fusion proteins and their good reactogenicity to the specific antibody. Moreover, we predicted the 3D structures of the partial antigen and fusion proteins through homology modeling using SWISS-MODEL, which displayed the relatively independent structures of M1 and HA2 proteins. (Figures 2D–F).

### The Growth Ability of the Recombinant *L. Lactis in vitro*

**Figure 3:**
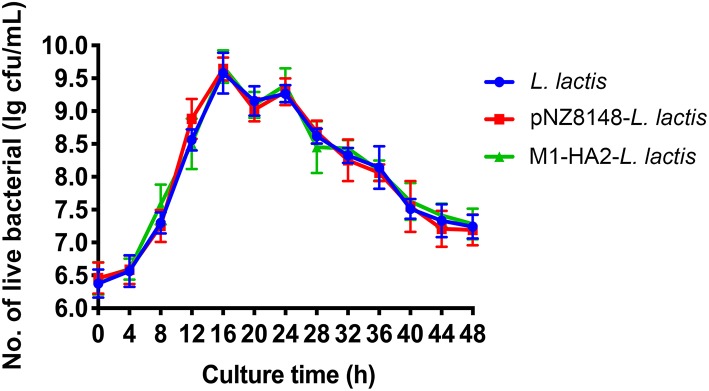
The growth curve of the *L. lactis* strain. The live bacteria counts were performed every 4 h, all data were expressed as means ± SD.

To verify the effect of the recombinant pNZ8148-M1-HA2 plasmid on the growth of *L. lactis*, we measured the growth curve (Figure 3). The results showed that the recombinant plasmid had no significant effect on the growth of *L. lactis* at different time points (*P* > 0.05).

### HI Antibody Levels Induced by M1-HA2-*L. Lactis* Strain in Chickens

**Figure 4:**
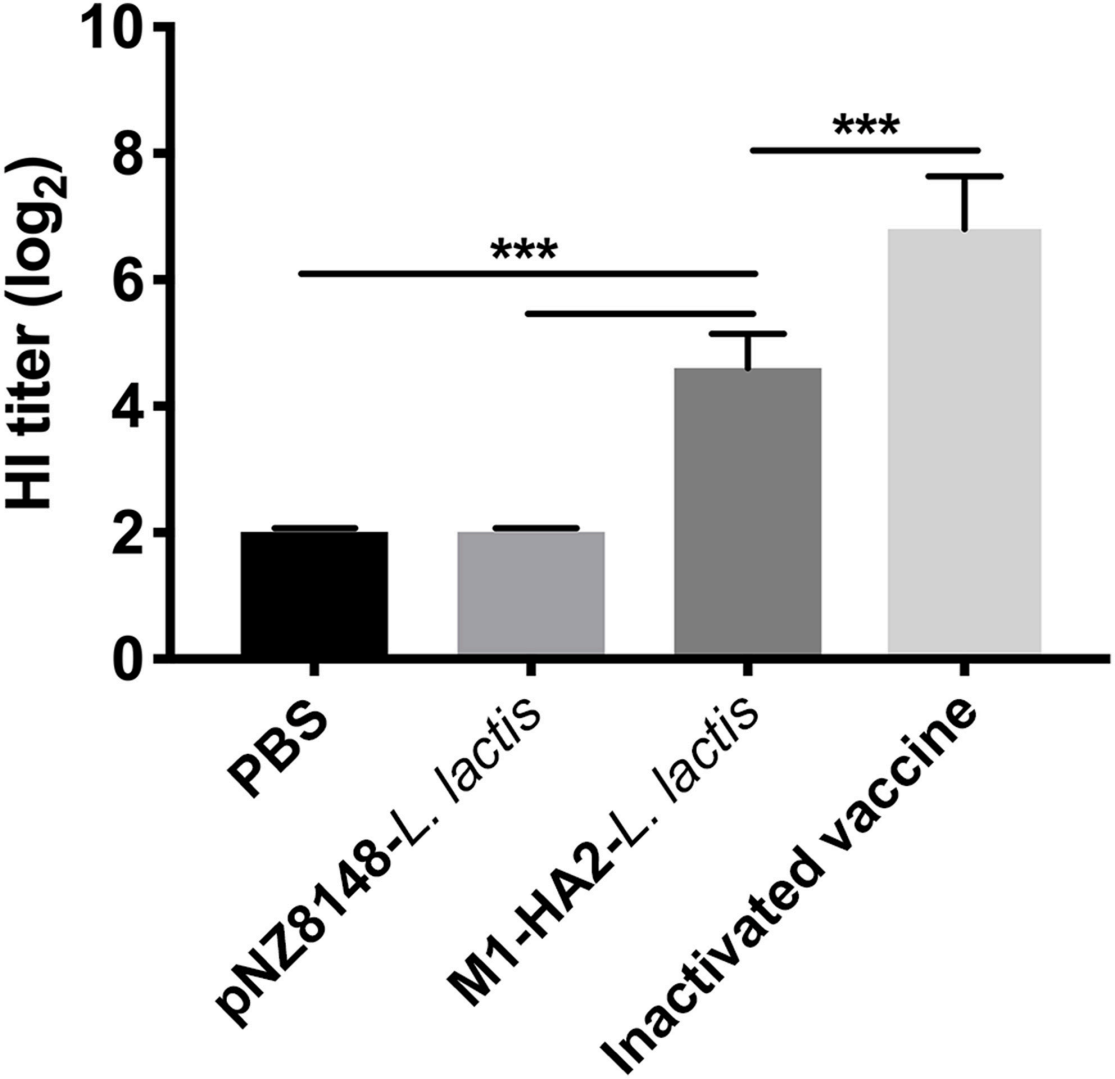
The HI result of chickens after vaccination. Antibody titers determined by employing an HI assay at 1 week after the boosting vaccination. HI titers were evaluated using an HI assay with H9N2 AIV and samples. The bars represent the means ± SD for the group (*n* = 5). Significant values (***P < 0.001) are shown by an asterisk.

The recombinant M1-HA2 *L. lactis* strain was used as an oral vaccine to immunize SPF chickens. HI antibody levels, induced against HA2 in the fusion protein, were quantitated. Sera were collected from chickens at 21 dpv, and HI antibody titers were determined (Figure 4). HI antibody levels in chickens orally vaccinated with M1-HA2 *L. lactis* increased compared with chickens vaccinated with pNZ8148 *L. lactis* or PBS (*P* < 0.001 and *P* < 0.001, respectively). However, immunization with the inactivated vaccine induced higher HI antibody levels than the recombinant M1-HA2 *L. lactis* oral vaccine in the normal dose range (*P* < 0.001).

### Detection of Mucosal SIgA and Serum IgG

**Figure 5:**
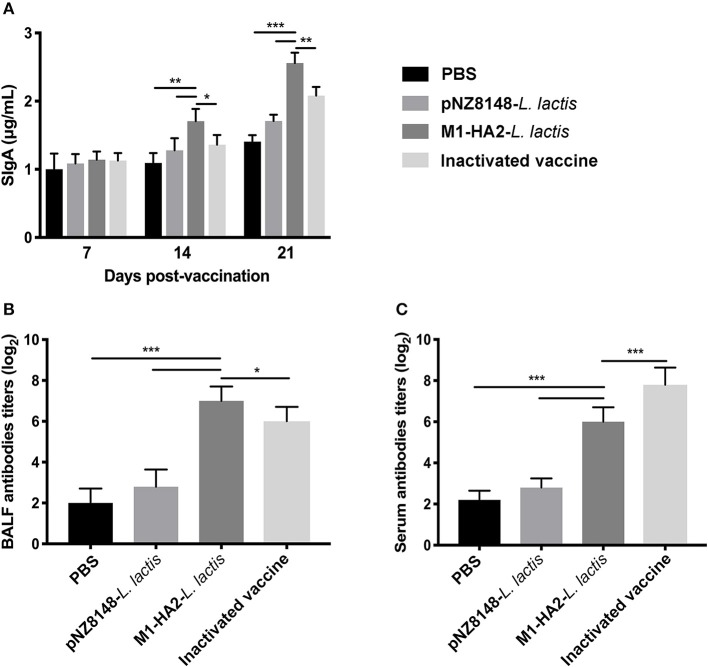
Mucosal antibody levels were measured at different time points. **(A)** Total SIgA (*n* = 5/group) in feces was assessed by ELISA at 7, 14, and 21 dpv. **(B)** Specific SIgA antibody titer in BALF. **(C)** Specific IgG antibody titer in serum was measured by ELISA. The bars represent the means ± SD for the group (*n* = 5). Significant values (*P < 0.05, **P < 0.01, and ***P < 0.001) are shown by an asterisk.

Levels of SIgA in intestinal mucosa and bronchoalveolar lavage fluid (BALF) following vaccination were measured using the Chicken SIgA ELISA Kit. Levels of serum IgG were measured by indirect ELISA. The SIgA content in each group showed a continuous upward trend. At 21 dpv, Intestinal SIgA levels in the M1-HA2 *L. lactis*-vaccinated group reached 2.70 μg/mL, 1.92-fold higher than those of the PBS-vaccinated group and 1.7-fold higher than those of the pNZ8148 *L. lactis*-vaccinated group (*P* < 0.001 and *P* < 0.001, respectively). SIgA levels in the M1-HA2 *L. lactis*-vaccinated group were also significantly higher compared with the inactivated vaccine group (*P* < 0.01) (Figure 5A). Antigen-specific SIgA titers in BALF showed a similar trend to antigen-specific serum IgG titers. Titers in the M1-HA2 *L. lactis*-vaccinated group were significantly higher than those in the PBS-vaccinated and pNZ8148 *L. lactis*-vaccinated groups (*P* < 0.001 and *P* < 0.001), respectively. However, SIgA and serum IgG titers differed significantly between the M1-HA2 *L. lactis*-vaccinated group and the inactivated vaccine group (*P* < 0.05 and *P* < 0.001, respectively) (Figures 5B,C).

### Real-Time PCR Assay for Detection of Intestinal Mucosal Cytokines

**Figure 6:**
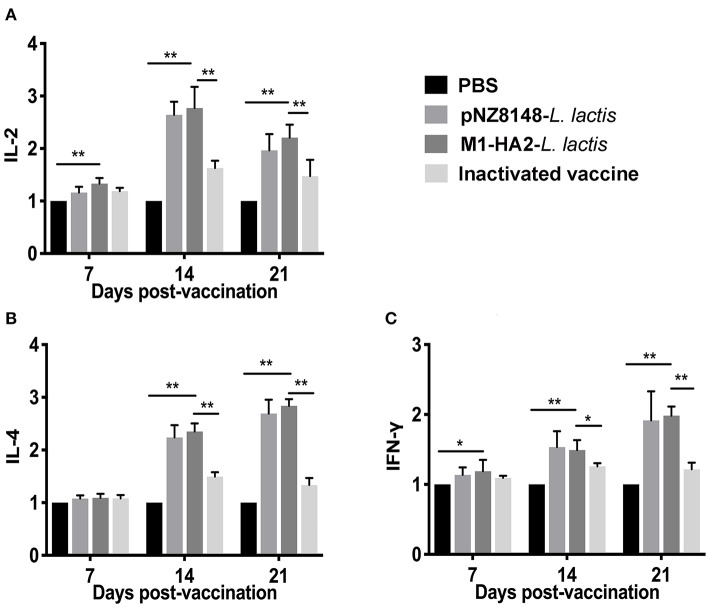
Changes of cytokines in chickens. Chickens in four groups were vaccinated with M1-HA2-*L. lactis*, pNZ8148-*L. lactis*, inactivated vaccine, and PBS, respectively. Intestinal tissues were collected at 7, 14, and 21 dpv. Then IL-2 **(A)**, IL-4 **(B)**, and IFN-γ **(C)** were detected via real-time PCR. *Indicates statistical significance at *P* < 0.05; ** represents *P* < 0.01. All values shown are means ± SD from five independent experiments.

At 7 dpv, levels of interleukin (IL)-2 and interferon (IFN)-γ in the M1-HA2 *L. lactis*-vaccinated group were significantly higher than those in the PBS-vaccinated group (*P* < 0.01 and *P* < 0.05, respectively). There were no significant differences in IL-2 and IFN-γ levels among the other groups (*P* > 0.05). At 14 dpv, levels of IL-2, IL-4 and IFN-γ in the M1-HA2 *L. lactis*-vaccinated group were 2.77-, 2.35-, and 1.48-fold higher than those in the PBS-vaccinated group (*P* < 0.01). However, levels of these cytokines did not differ significantly between the M1-HA2 *L. lactis*-vaccinated group and the pNZ8148 *L. lactis*-vaccinated group (*P* > 0.05). After 3 weeks, the M1-HA2 *L. lactis*-vaccinated group showed significantly increased levels of all three cytokines compared with the PBS-vaccinated and inactivated vaccine groups (*P* < 0.01) (Figures 6A–C).

### Cell-Mediated Immune Response Induced by M1-HA2-*L. lactis* Strain in Chickens

The percentage of CD4^+^ and CD8^+^ T cells directly reflects the immune function of animals (25). The results of statistical analysis of CD4^+^ and CD8^+^ T lymphocytes in peripheral blood are shown in Table 3, 4, respectively. Compared with the PBS group, the CD4^+^ levels in the other groups were significantly different at 21 dpv (*P* < 0.05). Similarly, the CD8^+^ levels of the pNZ8148-*L. lactis* and the M1-HA2-*L. lactis* groups were significantly higher than the other two groups (*P* < 0.05). The inactivated vaccine group was significantly higher than the PBS group (*P* < 0.05).

**Table 3:** Changes of CD4^+^ T lymphocyte counts in the peripheral blood.

**Group**	Days post–vaccination^*^			
	**0**	**7**	**14**	**21**
PBS	16.90 ± 0.26^a^	18.47 ± 0.45^a^	19.27 ± 0.72^a^	20.03 ± 0.67^a^
pNZ8148-*L. lactis*	17.53 ± 0.40^b^	20.27 ± 0.38^b^	22.13 ± 0.25^b^	27.07 ± 0.40^b^
M1-HA2-*L. lactis*	17.30 ± 0.20^b^	20.97 ± 0.50^b^	23.87 ± 0.32^c^	28.30 ± 0.36^c^
H9N2 inactivated vaccine	17.83 ± 0.25^b^	18.77 ± 0.15^a^	19.47 ± 0.45^a^	22.37 ± 0.45^d^

* *Different lowercase letters in the same column represent significant differences at the same dpv (P < 0.05). Data are expressed as percentage ± SD*.

**Table 4:** Changes of CD8^+^ T lymphocyte counts in the peripheral blood.

**Group**	Days post–vaccination^*^			
	**0**	**7**	**14**	**21**
PBS	10.87 ± 0.80^a^	11.17 ± 0.67^a^	11.93 ± 0.46^a^	12.13 ± 0.32^a^
pNZ8148-*L. lactis*	11.80 ± 0.62^a^	12.77 ± 0.25^b^	13.40 ± 0.45^b^	14.30 ± 0.36^b^
M1-HA2-*L. lactis*	11.43 ± 0.41^a^	13.33 ± 0.55^b^	13.63 ± 0.35^b^	14.57 ± 0.25^b^
H9N2 inactivated vaccine	11.68 ± 0.65^a^	11.27 ± 0.40^a^	12.03 ± 0.55^a^	12.77 ± 0.15^c^

* *Different lowercase letters in the same column represent significant differences at the same dpv (P < 0.05). Data are expressed as percentage ± SD*.

### Protective Effect of M1-HA2-*L. Lactis* Strain on H9N2 Subtype of AIV

**Figure 7:**
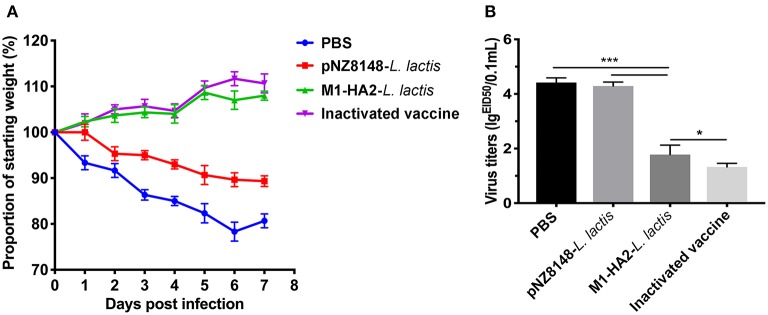
Protective effect of recombinant M1-HA2-*L. lactis* against H9N2 AIV challenge. **(A)** Weight loss (%) of the vaccinated chickens after challenge with AIV. **(B)** The virus titers of lung samples from vaccinated chickens were obtained at 7 days after challenge and infectivity was measured by EID_50_. The bars represent the means ± SD for each group (*n* = 5). Significance values (*P < 0.05, ***P < 0.001) are shown by an asterisk.

In order to evaluate the protective effect of recombinant M1-HA2-*L. lactis* strain on the H9N2 subtype AIV, the body weight changes and lung tissue virus titers of each group were statistically analyzed after the challenge experiment. The results indicated that the body weights of the PBS group and the pNZ8148-*L. lactis* group showed downward trends after H9N2 virus infection, and both were extremely significantly lower than the other two groups (*P* < 0.001 and *P* < 0.001). However, there was no significant difference between the M1-HA2-*L. lactis* group and the inactivated vaccine group (*P* > 0.05) (Figure 7A). In addition, we detected the virus titers in lung of each group at 7 days after infection. The results showed that the M1-HA2-*L. lactis* group was significantly higher than the inactivated vaccine group (*P* < 0.05), but compared with the PBS and pNZ8148-*L. lactis* groups, the lung virus titer was significantly reduced (*P* < 0.001 and *P* < 0.001) (Figure 7B).

### Histopathological Observation of Lungs

**Figure 8:**
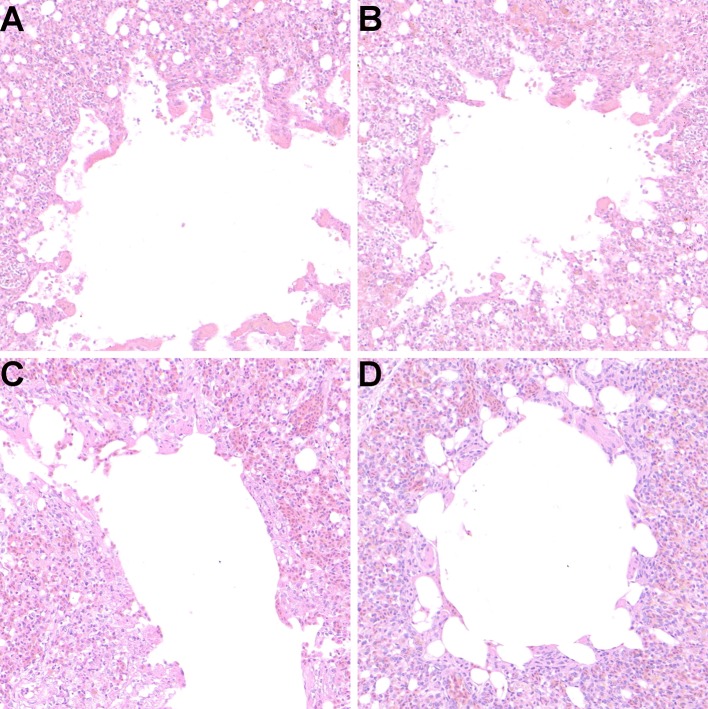
Protective effect of recombinant M1-HA2-*L. lactis* on lung injury induced by AIV infection (10^6.0^ EID_50_). **(A)** PBS, **(B)** pNZ8148-*L. lactis*, **(C)** M1-HA2-*L. lactis*, **(D)** H9N2 inactivated vaccine. Seven days after challenge with AIV, lung tissue of each group was evaluated by histopathological analysis. The sections were stained with H&E.

In order to further confirm the protective effect of the oral vaccine, the pulmonary sections of each group were prepared and evaluated pathological changes (Figures 8A–D). The results showed that there were no obvious lesions in the M1-HA2-*L. lactis* group and the inactivated vaccine group. The other groups had different degrees of lesions, which the homogeneous red-stained protein exuded a large number of inflammatory cells, the lung chamber collapsed, and the pulmonary epithelial cells detached.

## Discussion

The prevention and control of H9N2 avian influenza is a serious problem worldwide. Currently, the most common strategy is to inoculate with inactivated vaccines for almost all poultry in China. However, the H9N2 AIV is still prevalent in China, although vaccine strains are constantly being updated. It is likely difficult to effectively control the spread of H9N2 AIVs using inactivated vaccines that mainly induce humoral immunity; thus, promoting mucosal immune responses may contribute to the control on H9N2 virus. In this study, we constructed a recombinant *L. lactis* strain, which secreted a M1-HA2 fusion protein of the H9N2 virus. The evaluation of the immune index showed that it induced substantial mucosal immunity and a good protective effect against virus challenge.

The mucosa is the first physical barrier against the entry of pathogenic microorganisms into animals, and mucosal immune is the primary protection to microbial infection. The H9N2 initially enters the body through the respiratory mucosa; therefore, mucosal immunity must be an important and effective way to fight against H9N2 virus. The SIgA immunoglobulin is an important component of mucosal immunity (26). It can form a protective barrier by adhesion to epithelial cells and can also adhere to the newly synthesized viral proteins in the infected cells then interfere with the virion assembly. The protective potential of the SIgA antibody appears to be the result of its polymeric nature, resulting in a higher affinity of the antibody for AIV and preventing infection by blocking adhesion to epithelial cells. Our results indicated that the constructed recombinant *L. lactis* could secrete and express M1-HA2 fusion protein and induce substantial production of SIgA. After virus challenge, our results showed that recombinant *L. lactis*-immunized chickens induced immune protection, which was consistent with the results of inactivated vaccines, significantly inhibiting chicken weight loss, histopathological damage, and inflammatory response. In fact, the inactivated vaccine induced higher serum antibodies, which may be due to its higher antigen content and the effect of the vaccine adjuvant, but the recombinant *L. lactis* oral vaccine induced higher mucosal antibodies. Thus, the recombinant *L. lactis* can be used as an oral vaccine to compensate for functional deficiencies in inactivated vaccines. Moreover, the probiotic effect of *L. lactis* promoted cellular response and we also detected an up-regulation of IL-2, IL-4, and IFN-γ cytokines which promoted the activation and proliferation of the intestinal mucosal lymphoid B cells and induced the secretion of specific SIgA. They participated in the transmission of information and played an important role in the biological processes of the organisms (27).

As described above, the M1-HA2 fusion protein was expressed in *L. lactis*. The M1 protein, one of the most abundant proteins in virions, is relatively conserved. It may affect viral replication during early viral infection, mediating germination and assembly of progeny virions (28). Hemagglutinin is a major target for neutralizing antibody agglutination. A subunit vaccine based on the conserved sequence HA2 induced a wide range of cross-reactive antibody responses and provided protection against the influenza virus (29, 30). Related studies have shown that the expression of M1 protein by *E. coli* can be used as a candidate protein for the development of novel influenza vaccines (31). Previous studies have clarified the immune effects of M1 protein and the production of specific serum antibodies against H9N2 viruses (32). Mice immunized with purified M1 antigen and chitosan showed strong immune responses and were resistant to infection by multiple subtypes of influenza virus (33). In our study, we also selected the M1 protein, but due to its relatively small molecular mass, we fused it to another protective antigen, HA2. The immunogenicity of a recombinant *L. lactis* strain expressing this fusion protein was similar to that of a previously-reported *Lactobacillus* strain expressing H9N2 M1 protein (34). Previous reports have documented the immunoprotective effect of the HA2 protein. For instance, *Lactobacillus plantarum* expressing H9N2 HA2 could elicit anti-AIV humoral immunity and specific cell-mediated responses (35). Moreover, a M2e-HA2 fusion protein antigen elicited broader and more potent viral inhibition than either of the fusion partners alone (36). Another previous study showed that *L. plantarum* displaying an H9N2 M2e-HA2 fusion protein on the bacterial surface elicited protective immune responses against viral infection (37). Although M2e contains conserved neutralization epitopes, its low immunogenicity requires it to be fused with other proteins or other high-molecular weight carriers to generate an immune response. The M1 protein used in our study is a highly conserved structural protein and has been used as a potential candidate for the design of cross-protective vaccines. Additionally, the M1 and HA2 genes used in this study were derived from a clinical H9N2 isolate which was selected based on its recognition by multiple sera. Moreover, the M1 and HA2 antigens were structurally separated by a linker sequence, theoretically allowing them to play distinct antigenic roles. Additionally, we added a secretory peptide gene to the expression vector, which resulted in secretion of the fusion protein from *L. lactis*. Therefore, our design has several improvements over previous studies.

The most significant advantage of oral vaccines is the ability to trigger mucosal and systemic immune responses against foreign microbial infections (38). Mucosal immunization leads to stronger mucosal immune responses than immunization by other routes (39). This is important for respiratory tract pathogens, like the influenza virus. *L. lactis*, as a probiotic, is an ideal oral live vaccine antigen delivery vector, which has many advantages over traditional vaccines. It stimulates the local immune response in the intestine while exerting probiotic effects on the animal body, thus improving the mucosal immunity and resisting colonization of certain pathogenic bacteria. This method is simple to operate, in compliance with the animal welfare regulations, and suitable for large-scale animal vaccination. In this study, a recombinant *L. lactis* strain expressing an H9N2 M1-HA2 fusion protein was successfully constructed. Oral vaccination with this strain could significantly reduce H9N2 infection in chickens and activate mucosal immunity. Our findings provide a feasible method for mucosal prevention and control of H9N2 subtype AIV. The combined application of oral vaccines and inactivated vaccines may achieve superior efficacy.

## Data Availability Statement

All datasets generated for this study are included in the article/supplementary material.

## Ethics Statement

The animal study was reviewed and approved by Animal Protection and Utilization Committee of Shandong Agricultural University (Permit number: 20010510).

## Author Contributions

RZ, KW, HH, ZS, and HS designed research. ZS, HS, YM, JH, XN, RC, and LH performed research. ZS, HS, HH, KW, and RZ analyzed data. ZS, HS, KW, and RZ wrote the paper.

### Conflict of Interest

The authors declare that the research was conducted in the absence of any commercial or financial relationships that could be construed as a potential conflict of interest.
